# P-2236. Performance Characteristics of Procalcitonin in the Inpatient Setting

**DOI:** 10.1093/ofid/ofae631.2389

**Published:** 2025-01-29

**Authors:** Albert Park, Trisha S Nakasone, Amit Kaushal, Cybele Renault

**Affiliations:** Stanford University School of Medicine, San Mateo, California; Veterans Affairs Palo Alto Health Care System, Palo Alto, California; Veterans Affairs Palo Alto Health Care System, Palo Alto, California; Veterans Affairs Palo Alto Health Care System, Palo Alto, California

## Abstract

**Background:**

Procalcitonin (PCT) is a marker used in clinical decision-making for suspected bacterial infections. While its efficacy in guiding antibiotic de-escalation when used as a trend is well-established, its use as a stand-alone value is prevalent in both ICU and non-ICU settings despite the absence of evidence-based guidelines. Our study evaluates the performance of PCT as individual values and aims to discern organisms associated with true positive or false negative PCT values.

**Figure 1.**
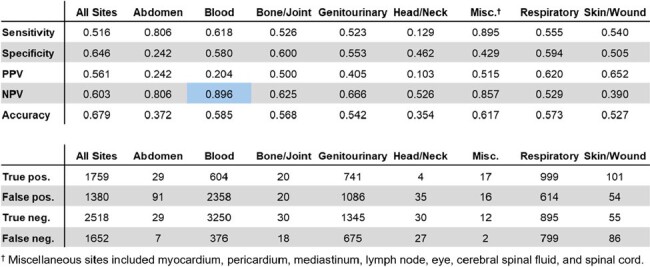
Performance characteristics of PCT by site of infection

**Methods:**

We performed a retrospective cohort study of patients at the Palo Alto Veterans Affairs Medical Center who underwent PCT testing from 1/1/2020 to 9/28/2023. We identified cultures drawn within 3 days of each PCT value to characterize the diagnostic performance of individual PCT values. Subsequently, we excluded organisms identified within 1 week of each other and performed one-sample z-tests for proportions to identify organisms associated with true positive (TP) or false negative PCT values based on site of infection.

**Figure 2.**
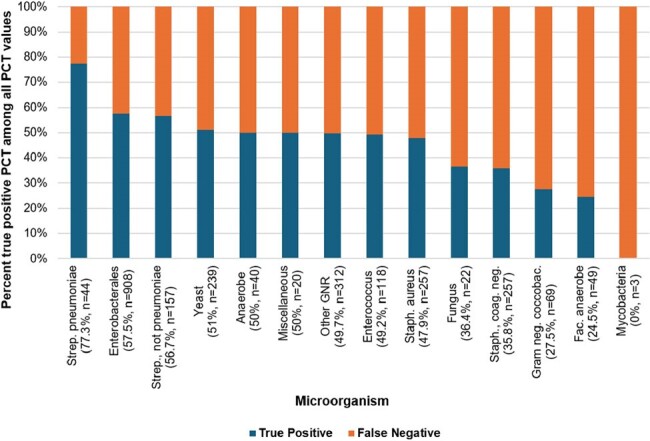
Microorganisms associated with true positive vs. false negative PCT from all culture sites

**Results:**

3204 patients underwent PCT testing during the study period and 4027 cultures were drawn. Blood culture PCT sensitivity was 0.618, specificity 0.580, positive predictive value (PPV) 0.204, and negative predictive value (NPV) 0.896 (Figure 1). Respiratory culture PCT sensitivity was 0.555, specificity 0.594, PPV 0.620, and NPV 0.529. 2495 organisms separated by at least 1 week from each other were identified by culture. *Enterobacterales* (TP rate 82.2%), *Staphylococcus aureus* (TP rate 81.6%), *Streptococcus pneumoniae* (TP rate 84.6%), and non-pneumococcal *Strep.* (TP rate 74.1%) were strongly associated with positive PCT values in blood cultures (Figures 2 and 3). *Enterobacterales* (TP rate 63.1%) and *Strep. pneumoniae* (TP rate 74.2%) were strongly associated with positive PCT values in respiratory cultures.

**Figure 3.**
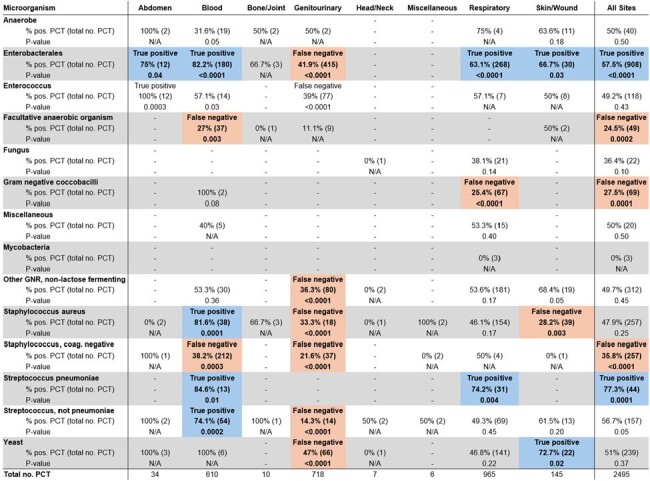
Microorganisms associated with true positive vs. false negative PCT by site of infection

**Conclusion:**

While the NPV of individual PCT values is 89.6% for blood cultures, individual PCT values otherwise have limited diagnostic accuracy for active infections. Certain organisms show stronger associations with positive PCT values. Further investigation may offer insights into specific bacterial characteristics that drive PCT response in infected patients. However, in most cases, culture-identified organisms do not reliably correlate with positive PCT values.

**Disclosures:**

All Authors: No reported disclosures

